# Duration of mRNA vaccine protection against SARS-CoV-2 Omicron BA.1 and BA.2 subvariants in Qatar

**DOI:** 10.1038/s41467-022-30895-3

**Published:** 2022-06-02

**Authors:** Hiam Chemaitelly, Houssein H. Ayoub, Sawsan AlMukdad, Peter Coyle, Patrick Tang, Hadi M. Yassine, Hebah A. Al-Khatib, Maria K. Smatti, Mohammad R. Hasan, Zaina Al-Kanaani, Einas Al-Kuwari, Andrew Jeremijenko, Anvar Hassan Kaleeckal, Ali Nizar Latif, Riyazuddin Mohammad Shaik, Hanan F. Abdul-Rahim, Gheyath K. Nasrallah, Mohamed Ghaith Al-Kuwari, Adeel A. Butt, Hamad Eid Al-Romaihi, Mohamed H. Al-Thani, Abdullatif Al-Khal, Roberto Bertollini, Laith J. Abu-Raddad

**Affiliations:** 1 Infectious Disease Epidemiology Group, Weill Cornell Medicine-Qatar, Cornell University, Doha, Qatar; 2 World Health Organization Collaborating Centre for Disease Epidemiology Analytics on HIV/AIDS, Sexually Transmitted Infections, and Viral Hepatitis, Weill Cornell Medicine—Qatar, Qatar Foundation—Education City, Cornell University, Doha, Qatar; 3 Department of Population Health Sciences, Weill Cornell Medicine, Cornell University, New York, NY USA; 4 Mathematics Program, Department of Mathematics, Statistics, and Physics, College of Arts and Sciences, Qatar University, Doha, Qatar; 5 Hamad Medical Corporation, Doha, Qatar; 6 Biomedical Research Center, Member of QU Health, Qatar University, Doha, Qatar; 7 Wellcome-Wolfson Institute for Experimental Medicine, Queens University, Belfast, UK; 8 Department of Pathology, Sidra Medicine, Doha, Qatar; 9 Department of Biomedical Science, College of Health Sciences, Member of QU Health, Qatar University, Doha, Qatar; 10 Department of Public Health, College of Health Sciences, QU Health, Qatar University, Doha, Qatar; 11 Primary Health Care Corporation, Doha, Qatar; 12 Department of Medicine, Weill Cornell Medicine, Cornell University, New York, NY USA; 13 Ministry of Public Health, Doha, Qatar

**Keywords:** Viral infection, Epidemiology

## Abstract

SARS-CoV-2 Omicron BA.1 and BA.2 subvariants are genetically divergent. We conducted a matched, test-negative, case-control study to estimate duration of protection of the second and third/booster doses of mRNA COVID-19 vaccines against BA.1 and BA.2 infections in Qatar. BNT162b2 effectiveness was highest at 46.6% (95% CI: 33.4–57.2%) against symptomatic BA.1 and at 51.7% (95% CI: 43.2–58.9%) against symptomatic BA.2 infections in the first three months after the second dose, but declined to ~10% or below thereafter. Effectiveness rebounded to 59.9% (95% CI: 51.2–67.0%) and 43.7% (95% CI: 36.5–50.0%), respectively, in the first month after the booster dose, before declining again. Effectiveness against COVID-19 hospitalization and death was 70–80% after the second dose and >90% after the booster dose. mRNA-1273 vaccine protection showed similar patterns. mRNA vaccines provide comparable, moderate, and short-lived protection against symptomatic BA.1 and BA.2 Omicron infections, but strong and durable protection against COVID-19 hospitalization and death.

## Abstract (web summary)

The SARS-CoV-2 Omicron variant has subvariants with divergent properties but relative vaccine effectiveness has not been characterized. Here, the authors show that mRNA vaccine effectiveness is similar for the subvariants BA.1 and BA.2, with a decline three months after the second dose and increase after the booster.

## Introduction

**Fig. 1:**
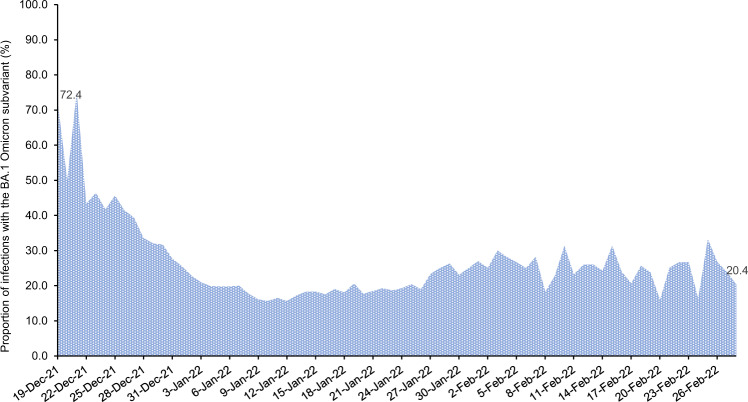
Distribution of SARS-CoV-2 BA.1 versus BA.2 Omicron infections. Proportion of Omicron infections with the BA.1 (versus BA.2) subvariant in PCR-positive tests assessed using TaqPath COVID-19 Combo Kit during the study period.

Qatar endured a severe acute respiratory syndrome coronavirus 2 (SARS-CoV-2) Omicron (B.1.1.529)^1^ wave that started on December 19, 2021 and peaked in mid-January, 2022^2–5^. The wave was first dominated by the BA.1 Omicron subvariant, but within a few days, the BA.2 subvariant predominated (Fig. 1). While BA.1 and BA.2 remain classified as subvariants of the Omicron variant, there is considerable genetic distance between them^6^. Accordingly, we investigated duration of protection of BNT162b2 (Pfizer-BioNTech)^7^ and mRNA-1273 (Moderna)^8^ mRNA coronavirus disease 2019 (COVID-19) vaccines, after the second dose and after the third/booster dose, against symptomatic BA.1 and BA.2 infections, between December 23, 2021 and February 28, 2022. Duration of vaccine protection was also investigated against any severe (acute-care hospitalization)^9^, critical (intensive-care-unit hospitalization)^9^, or fatal^10^ infection due to either Omicron subvariant.

Vaccine effectiveness was estimated using the test-negative, case–control study design^11,12^, applying methodology that was developed earlier to assess duration of protection of the BNT162b2^13^ and mRNA-1273^14^ vaccines in the same population during pre-Omicron SARS-CoV-2 infection waves (Methods). Cases (persons infected with BA.1, BA.2, or any-Omicron-subvariant) and controls (uninfected persons) were exact-matched by sex, 10-year-age group, nationality, and calendar week of polymerase chain reaction (PCR) test to control for established differences in the risk of exposure to SARS-CoV-2 infection in Qatar^15–19^.

## Results

### Main analyses

By February 28, 2022 (end of study), 1,308,926 individuals received 2 or more BNT162b2 doses, and 355,979 of these received a booster dose. Meanwhile, 894,142 individuals received 2 or more mRNA-1273 doses, and 146,961 of these received a booster dose. The median dates of first, second, and third doses were May 3, 2021, May 24, 2021, and December 27, 2021 for BNT162b2; and May 28, 2021, June 27, 2021, and January 16, 2022 for mRNA-1273, respectively. The median time between the first and second doses was 21 days (interquartile range (IQR), 21–22 days) for BNT162b2 and 28 days (IQR, 28–30 days) for mRNA-1273. The median time between the second and booster doses was 251 days (IQR, 233–275 days) for BNT162b2 and 236 days (IQR, 213–261 days) for mRNA-1273.

**Fig. 2:**
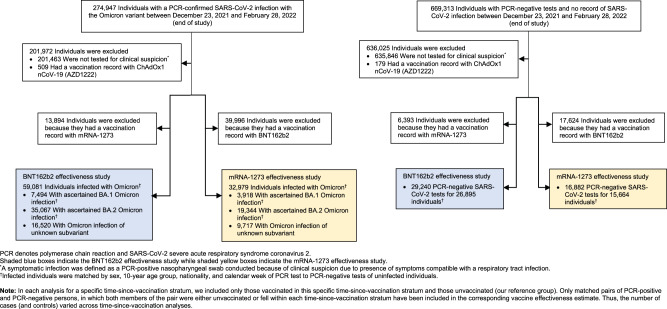
Study population selection process. Flowchart describing the population selection process for investigating effectiveness of the BNT162b2 and mRNA-1273 vaccines during the SARS-CoV-2 Omicron infection wave.

The process used to select the study populations is shown in Fig. 2. Demographic characteristics of the study populations are presented in Tables 1, 2. The study was conducted based on the total population of Qatar. The study populations are therefore representative of the internationally diverse, but predominantly young and male population of Qatar.

**Table 1:** Demographic characteristics of cases and controls in samples used to estimate effectiveness of the BNT162b2 vaccine against symptomatic SARS-CoV-2 BA.1 Omicron infection, symptomatic BA.2 Omicron infection, and any symptomatic Omicron infection.

Characteristics	Effectiveness against symptomatic SARS-CoV-2 BA.1 Omicron infection			Effectiveness against symptomatic SARS-CoV-2 BA.2 Omicron infection			Effectiveness against any symptomatic SARS-CoV-2 Omicron infection		
	Cases^a^ (PCR-positive)	Controls^a^ (PCR-negative)	SMD^b^	Cases^c^ (PCR-positive)	Controls^c^ (PCR-negative)	SMD^b^	Cases^d^ (PCR-positive)	Controls^d^ (PCR-negative)	SMD^b^
	*N* = 7022	*N* = 12,278		*N* = 21,541	*N* = 21,541		*N* = 39,855	*N* = 23,814	
Median age (IQR)—years	32 (22–42)	32 (20–42)	0.06^e^	32 (18–42)	31 (18–42)	0.01^e^	32 (19–42)	32 (19–43)	0.01^e^
Age group—no. (%)									
<10 years	670 (9.5)	1282 (10.4)	0.05	3157 (14.7)	3157 (14.7)	0.00	5581 (14.0)	3501 (14.7)	0.03
10–19 years	875 (12.5)	1611 (13.1)		2458 (11.4)	2458 (11.4)		4594 (11.5)	2640 (11.1)	
20–29 years	1385 (19.7)	2389 (19.5)		4016 (18.6)	4016 (18.6)		7344 (18.4)	4369 (18.4)	
30–39 years	1983 (28.2)	3487 (28.4)		5561 (25.8)	5561 (25.8)		10,419 (26.1)	6066 (25.5)	
40–49 years	1053 (15.0)	1782 (14.5)		2824 (13.1)	2824 (13.1)		5462 (13.7)	3254 (13.7)	
50–59 years	674 (9.6)	1115 (9.1)		2166 (10.1)	2166 (10.1)		3995 (10.0)	2440 (10.3)	
60–69 years	279 (4.0)	436 (3.6)		951 (4.4)	951 (4.4)		1685 (4.2)	1050 (4.4)	
70+ years	103 (1.5)	176 (1.4)		408 (1.9)	408 (1.9)		775 (1.9)	494 (2.1)	
Sex									
Male	3437 (49.0)	6335 (51.6)	0.05	11,986 (55.6)	11,986 (55.6)	0.00	21,951 (55.1)	13,257 (55.7)	0.01
Female	3585 (51.1)	5943 (48.4)		9555 (44.4)	9555 (44.4)		17,904 (44.9)	10,557 (44.3)	
Nationality^f^									
Bangladeshi	102 (1.5)	184 (1.5)	0.05	521 (2.4)	521 (2.4)	0.00	872 (2.2)	614 (2.6)	0.06
Egyptian	416 (5.9)	723 (5.9)		1384 (6.4)	1384 (6.4)		2360 (5.9)	1343 (5.6)	
Filipino	761 (10.8)	1357 (11.1)		2063 (9.6)	2063 (9.6)		3844 (9.6)	2227 (9.4)	
Indian	793 (11.3)	1467 (12.0)		3077 (14.3)	3077 (14.3)		5403 (13.6)	3314 (13.9)	
Nepalese	80 (1.1)	138 (1.1)		430 (2.0)	430 (2.0)		632 (1.6)	369 (1.6)	
Pakistani	152 (2.2)	279 (2.3)		788 (3.7)	788 (3.7)		1325 (3.3)	805 (3.4)	
Qatari	2824 (40.2)	5074 (41.3)		7277 (33.8)	7277 (33.8)		14,632 (36.7)	8304 (34.9)	
Sri Lankan	62 (0.9)	105 (0.9)		299 (1.4)	299 (1.4)		497 (1.3)	313 (1.3)	
Sudanese	328 (4.7)	576 (4.7)		1036 (4.8)	1036 (4.8)		1730 (4.3)	1026 (4.3)	
Other nationalities	1504 (21.4)	2375 (19.3)		4666 (21.7)	4666 (21.7)		8560 (21.5)	5499 (23.1)	

The table was generated by combining the matched samples of the various time-since-vaccination strata.

*IQR* interquartile range, *PCR* polymerase chain reaction, *SMD* standardized mean difference.

^a^Cases and controls were matched one-to-two by sex, 10-year-age group, nationality, and calendar week of PCR test.

^b^SMD is the difference in the mean of a covariate between groups divided by the pooled standard deviation. An SMD < 0.1 indicates adequate matching.

^c^Cases and controls were matched one-to-one by sex, 10-year-age group, nationality, and calendar week of PCR test.

^d^Cases and controls were matched two-to-one by sex, 10-year-age group, nationality, and calendar week of PCR test.

^e^SMD is for the mean difference between groups divided by the pooled standard deviation.

^f^Nationalities were chosen to represent the most populous groups in Qatar.

**Table 2:** Demographic characteristics of cases and controls in samples used to estimate effectiveness of the mRNA-1273 vaccine against symptomatic SARS-CoV-2 BA.1 Omicron infection, symptomatic BA.2 Omicron infection, and any symptomatic Omicron infection.

Characteristics	Effectiveness against symptomatic SARS-CoV-2 BA.1 Omicron infection			Effectiveness against symptomatic SARS-CoV-2 BA.2 Omicron infection			Effectiveness against any symptomatic SARS-CoV-2 Omicron infection		
	Cases^a^ (PCR-positive)	Controls^a^ (PCR-negative)	SMD^b^	Cases^c^ (PCR-positive)	Controls^c^ (PCR-negative)	SMD^b^	Cases^d^ (PCR-positive)	Controls^d^ (PCR-negative)	SMD^b^
	*N* = 3574	*N* = 6176		*N* = 13,537	*N* = 13,537		*N* = 21,810	*N* = 13,288	
Median age (IQR)—years	30 (15–38)	29 (11–38)	0.07^e^	28 (10–37)	28 (10–38)	0.01^e^	28 (9–37)	28 (9–38)	0.00^e^
Age group—no. (%)									
<10 years	670 (18.8)	1282 (20.8)	0.07	3149 (23.3)	3149 (23.3)	0.00	5576 (25.6)	3496 (26.3)	0.03
10–19 years	300 (8.4)	549 (8.9)		1475 (10.9)	1475 (10.9)		1692 (7.8)	993 (7.5)	
20–29 years	771 (21.6)	1286 (20.8)		2633 (19.5)	2633 (19.5)		4311 (19.8)	2608 (19.6)	
30–39 years	1037 (29.0)	1788 (29.0)		3427 (25.3)	3427 (25.3)		5692 (26.1)	3368 (25.4)	
40–49 years	475 (13.3)	797 (12.9)		1512 (11.2)	1512 (11.2)		2575 (11.8)	1568 (11.8)	
50–59 years	231 (6.5)	349 (5.7)		880 (6.5)	880 (6.5)		1346 (6.2)	853 (6.4)	
60–69 years	68 (1.9)	89 (1.4)		315 (2.3)	315 (2.3)		400 (1.8)	261 (2.0)	
70+ years	22 (0.6)	36 (0.6)		146 (1.1)	146 (1.1)		218 (1.0)	141 (1.1)	
Sex									
Male	1769 (49.5)	3232 (52.3)	0.06	7717 (57.0)	7717 (57.0)	0.00	12,678 (58.1)	7745 (58.3)	0.00
Female	1805 (50.5)	2944 (47.7)		5820 (43.0)	5820 (43.0)		9132 (41.9)	5543 (41.7)	
Nationality^f^									
Bangladeshi	74 (2.1)	132 (2.1)	0.07	443 (3.3)	443 (3.3)	0.00	762 (3.5)	547 (4.1)	0.06
Egyptian	224 (6.3)	393 (6.4)		897 (6.6)	897 (6.6)		1249 (5.7)	715 (5.4)	
Filipino	524 (14.7)	890 (14.4)		1402 (10.4)	1402 (10.4)		2396 (11.0)	1389 (10.5)	
Indian	535 (15.0)	1007 (16.3)		2256 (16.7)	2256 (16.7)		3719 (17.1)	2306 (17.4)	
Nepalese	74 (2.1)	132 (2.1)		431 (3.2)	431 (3.2)		625 (2.9)	363 (2.7)	
Pakistani	118 (3.3)	221 (3.6)		658 (4.9)	658 (4.9)		1042 (4.8)	633 (4.8)	
Qatari	866 (24.2)	1554 (25.2)		3,364 (24.9)	3364 (24.9)		5117 (23.5)	2955 (22.2)	
Sri Lankan	42 (1.2)	74 (1.2)		262 (1.9)	262 (1.9)		444 (2.0)	271 (2.0)	
Sudanese	212 (5.9)	385 (6.2)		789 (5.8)	789 (5.8)		1273 (5.8)	758 (5.7)	
Other nationalities	905 (25.3)	1388 (22.5)		3,035 (22.4)	3035 (22.4)		5183 (23.8)	3351 (25.2)	

The table was generated by combining the matched samples of the various time-since-vaccination strata.

*IQR* interquartile range, *PCR* polymerase chain reaction, *SMD* standardized mean difference.

^a^Cases and controls were matched one-to-two by sex, 10-year-age group, nationality, and calendar week of PCR test.

^b^SMD is the difference in the mean of a covariate between groups divided by the pooled standard deviation. An SMD < 0.1 indicates adequate matching.

^c^Cases and controls were matched one-to-one by sex, 10-year-age group, nationality, and calendar week of PCR test.

^d^Cases and controls were matched two-to-one by sex, 10-year-age group, nationality, and calendar week of PCR test.

^e^SMD is for the mean difference between groups divided by the pooled standard deviation.

^f^Nationalities were chosen to represent the most populous groups in Qatar.

**Fig. 3:**
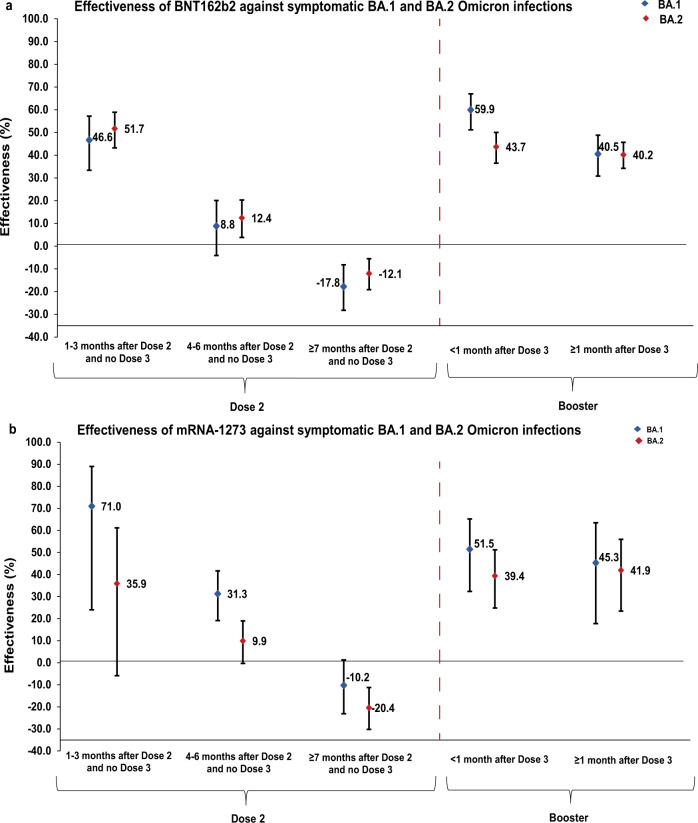
Effectiveness of mRNA vaccines against symptomatic SARS-CoV-2 BA.1 and BA.2 Omicron infections. Effectiveness (**a**) of the BNT162b2 vaccine and (**b**) of the mRNA-1273 vaccine. **a** Includes 7022 and 12,278 biologically independent samples for cases and controls, respectively, in the BA.1 analysis and 21,541 biologically independent samples for each of cases and controls in the BA.2 analysis in the BNT162b2 vaccine study. **b** includes 3574 and 6176 biologically independent samples for cases and controls, respectively, in the BA.1 analysis and 13,537 biologically independent samples for each of cases and controls in the BA.2 analysis in the mRNA-1273 vaccine study. Data are presented as effectiveness point estimates. Error bars indicate the corresponding 95% confidence intervals.

BNT162b2 effectiveness against symptomatic BA.1 infection was highest at 46.6% (95% confidence interval (CI): 33.4–57.2%) in the first 3 months after the second dose, but then declined to ~10% or below thereafter (Fig. 3a and Table 3). Effectiveness rapidly rebounded to 59.9% (95% CI: 51.2–67.0%) in the first month after the booster dose, but then declined to 40.5% (95% CI: 30.8–48.8%) in the second month and thereafter. A similar pattern was observed for mRNA-1273 effectiveness (Fig. 3b and Table 4).

**Table 3:** Effectiveness of the BNT162b2 vaccine against symptomatic SARS-CoV-2 BA.1 Omicron infection, BA.2 Omicron infection, and any Omicron infection^a^.

Sub-studies^b^	Cases (PCR-positive)		Controls (PCR-negative)		Effectiveness in % (95% CI)^c^
	Vaccinated	Unvaccinated	Vaccinated	Unvaccinated	
Effectiveness against symptomatic BA.1 Omicron infection^d^					
Dose 1					
0–13 days after Dose 1 and no Dose 2	10	1969	20	3456	23.5 (−70.6 to 65.7)
≥14 days after Dose 1 and no Dose 2	25	1968	66	3441	39.2 (2.3 to 62.1)
Dose 2					
1–3 months after Dose 2 and no Dose 3	130	1992	376	3409	46.6 (33.4 to 57.2)
4–6 months after Dose 2 and no Dose 3	502	2004	941	3506	8.8 (−4.1 to 20.1)
≥7 months after Dose 2 and no Dose 3	3570	2060	6007	3947	−17.8 (−28.2 to −8.2)
Dose 3 (booster dose)					
<1 month after Dose 3	180	2008	622	3339	59.9 (51.2 to 67.0)
≥1 month after Dose 3	483	2031	1145	3441	40.5 (30.8 to 48.8)
Effectiveness against symptomatic BA.2 Omicron infection^e^					
Dose 1					
0–13 days after Dose 1 and no Dose 2	32	5744	34	5742	5.9 (−52.5 to 41.9)
≥14 days after Dose 1 and no Dose 2	64	5774	99	5739	36.1 (12.1 to 53.5)
Dose 2					
1–3 months after Dose 2 and no Dose 3	263	5964	496	5731	51.7 (43.2 to 58.9)
4–6 months after Dose 2 and no Dose 3	1203	5924	1318	5809	12.4 (3.8 to 20.3)
≥7 months after Dose 2 and no Dose 3	8003	5840	7762	6081	−12.1 (−19.1 to −5.5)
Dose 3 (booster dose)					
<1 month after Dose 3	709	6038	1034	5713	43.7 (36.5 to 50.0)
≥1 month after Dose 3	1580	6211	2029	5762	40.2 (34.2 to 45.7)
Effectiveness against any symptomatic Omicron infection^f^					
Dose 1					
0–13 days after Dose 1	56	12,174	34	7278	9.5 (−39.8 to 41.3)
≥14 days after Dose 1 and no Dose 2	151	12,205	130	7276	31.4 (12.5 to 46.3)
Dose 2					
1–3 months after Dose 2 and no Dose 3	585	12,623	599	7309	47.8 (40.8 to 53.9)
4–6 months after Dose 2 and no Dose 3	2479	12,590	1605	7333	16.3 (9.7 to 22.5)
≥7 months after Dose 2 and no Dose 3	16,435	12,564	9073	7637	−9.0 (−14.5 to −3.7)
Dose 3 (booster dose)^g^					
1 week after Dose 3	374	12,200	260	7304	17.7 (2.5 to 30.6)
2–3 weeks after Dose 3	566	12,524	662	7284	55.5 (49.3 to 61.0)
4–5 weeks after Dose 3	645	12,548	706	7283	51.5 (45.0 to 57.2)
6–7 weeks after Dose 3	866	12,542	770	7319	43.6 (36.5 to 49.9)
8–9 weeks after Dose 3	493	12,298	418	7320	31.5 (20.3 to 41.1)
10–11 weeks after Dose 3	331	12,296	310	7305	37.3 (25.4 to 47.3)
12–13 weeks after Dose 3	261	12,234	228	7295	32.6 (17.8 to 44.8)
≥14 weeks after Dose 3	446	12,231	358	7333	21.9 (7.7 to 33.9)

*CI* confidence interval, *PCR* polymerase chain reaction.

^a^A symptomatic infection was defined as a PCR-positive nasopharyngeal swab conducted because of clinical suspicion due to presence of symptoms compatible with a respiratory tract infection.

^b^In each analysis for a specific time-since-vaccination stratum, we included only those vaccinated in this specific time-since-vaccination stratum and those unvaccinated. Only matched pairs of PCR-positive and PCR-negative persons, in which both members of the pair were either unvaccinated or fell within each time-since-vaccination stratum have been included in the corresponding vaccine effectiveness estimate. Thus, the number of cases (and controls) varied across time-since-vaccination analyses.

^c^Vaccine effectiveness was estimated using the test-negative, case–control study design^11,12^.

^d^Cases and controls were matched one-to-two by sex, 10-year-age group, nationality, and calendar week of PCR test.

^e^Cases and controls were matched one-to-one by sex, 10-year age group, nationality, and calendar week of PCR test.

^f^Cases and controls were matched two-to-one by sex, 10-year-age group, nationality, and calendar week of PCR test.

^g^To assess booster effectiveness over longer time interval, the analysis for effectiveness against any symptomatic Omicron infection was subsequently extended until April 11, 2022. This extended analysis was done for only effectiveness against any symptomatic Omicron infection to optimize statistical precision with the larger case numbers.

**Table 4:** Effectiveness of the mRNA-1273 vaccine against symptomatic SARS-CoV-2 BA.1 Omicron infection, BA.2 Omicron infection, and any Omicron infection^a^.

Sub-studies^b^	Cases (PCR-positive)		Controls (PCR-negative)		Effectiveness in % (95% CI)^c^
	Vaccinated	Unvaccinated	Vaccinated	Unvaccinated	
Effectiveness against symptomatic BA.1 Omicron infection^d^					
Dose 1					
0–13 days after Dose 1 and no Dose 2	3	1942	8	3400	50.0 (−91.3 to 86.9)
≥14 days after Dose 1 and no Dose 2	14	1942	19	3405	−16.8 (−137.8 to 42.6)
Dose 2					
1–3 months after Dose 2 and no Dose 3	6	1943	27	3396	71.0 (24.0 to 89.0)
4–6 months after Dose 2 and no Dose 3	289	1976	667	3377	31.3 (19.1 to 41.7)
≥7 months after Dose 2 and no Dose 3	1125	1999	1847	3638	−10.2 (−23.1 to 1.3)
Dose 3 (booster dose)					
<1 month after Dose 3	55	1951	182	3377	51.5 (32.3 to 65.2)
≥1 month after Dose 3	36	1953	102	3396	45.3 (17.8 to 63.5)
Effectiveness against symptomatic BA.2 Omicron infection^e^					
Dose 1					
0–13 days after Dose 1 and no Dose 2	8	5651	10	5649	20.0 (−102.7 to 68.4)
≥14 days after Dose 1 and no Dose 2	31	5645	27	5649	−15.4 (−95.1 to 31.8)
Dose 2					
1–3 months after Dose 2 and no Dose 3	26	5664	40	5650	35.9 (−5.9 to 61.2)
4–6 months after Dose 2 and no Dose 3	989	5756	1059	5686	9.9 (−0.3 to 19.0)
≥7 months after Dose 2 and no Dose 3	2917	5627	2686	5858	−20.4 (−30.2 to −11.2)
Dose 3 (booster dose)					
<1 month after Dose 3	164	5727	250	5641	39.4 (24.8 to 51.2)
≥1 month after Dose 3	92	5709	149	5652	41.9 (23.4 to 56.0)
Effectiveness against any symptomatic Omicron infection^f^					
Dose 1					
0–13 days after Dose 1 and no Dose 2	17	11,987	11	7153	9.8 (−94.1 to 58.1)
≥14 days after Dose 1 and no Dose 2	52	11,984	36	7150	9.5 (−39.9 to 41.5)
Dose 2					
1–3 months after Dose 2 and no Dose 3	47	12,014	51	7151	43.2 (15.0 to 62.1)
4–6 months after Dose 2 and no Dose 3	1863	12,321	1294	7205	18.7 (11.3 to 25.5)
≥7 months after Dose 2 and no Dose 3	5820	12,144	3112	7374	−13.7 (−21.3 to −6.6)
Dose 3 (booster dose)^g^					
1 week after Dose 3	100	11,912	73	7132	19.7 (−9.7 to 41.2)
2–3 weeks after Dose 3	151	12,038	182	7126	53.7 (41.5 to 63.3)
4–5 weeks after Dose 3	109	12,000	135	7131	53.7 (39.6 to 64.6)
≥6 weeks after Dose 3	124	11,963	113	7134	34.9 (14.6 to 50.4)

*CI* confidence interval, *PCR* polymerase chain reaction.

^a^A symptomatic infection was defined as a PCR-positive nasopharyngeal swab conducted because of clinical suspicion due to presence of symptoms compatible with a respiratory tract infection.

^b^In each analysis for a specific time-since-vaccination stratum, we included only those vaccinated in this specific time-since-vaccination stratum and those unvaccinated. Only matched pairs of PCR-positive and PCR-negative persons, in which both members of the pair were either unvaccinated or fell within each time-since-vaccination stratum have been included in the corresponding vaccine effectiveness estimate. Thus, the number of cases (and controls) varied across time-since-vaccination analyses.

^c^Vaccine effectiveness was estimated using the test-negative, case–control study design^11,12^.

^d^Cases and controls were matched one-to-two by sex, 10-year-age group, nationality, and calendar week of PCR test.

^e^Cases and controls were matched one-to-one by sex, 10-year-age group, nationality, and calendar week of PCR test.

^f^Cases and controls were matched two-to-one by sex, 10-year-age group, nationality, and calendar week of PCR test.

^g^To assess booster effectiveness over longer time interval, the analysis for effectiveness against any symptomatic Omicron infection was subsequently extended until April 11, 2022. This extended analysis was done for only effectiveness against any symptomatic Omicron infection to optimize statistical precision with the larger case numbers.

BNT162b2 effectiveness against symptomatic BA.2 infection was highest at 51.7% (95% CI: 43.2–58.9%) in the first 3 months after the second dose, but then declined to ~10% or below thereafter (Fig. 3a and Table 3). Effectiveness rapidly rebounded to 43.7% (95% CI: 36.5–50.0%) in the first month after the booster dose and was 40.2% (95% CI: 34.2–45.7%) in the second month and thereafter. A similar pattern was observed for mRNA-1273 effectiveness (Fig. 3b and Table 4).

**Fig. 4:**
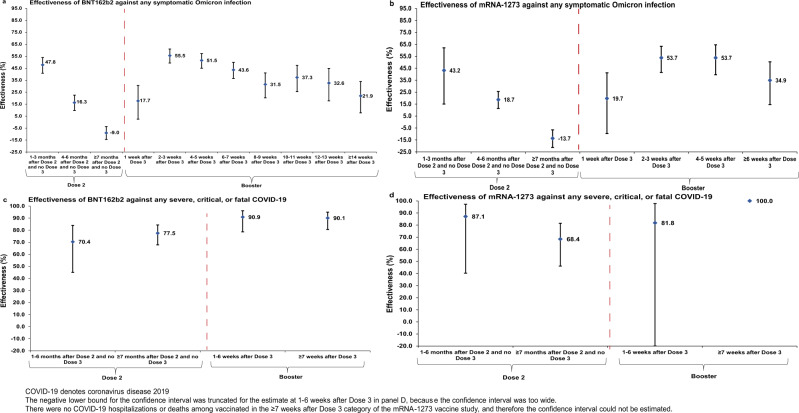
Effectiveness of mRNA vaccines against any symptomatic SARS-CoV-2 Omicron infection regardless of subvariant and against severe COVID-19. Effectiveness of the BNT162b2 and mRNA-1273 vaccines against any symptomatic SARS-CoV-2 Omicron infection regardless of subvariant (panels a and b, respectively) and against any severe^9^, critical^9^, or fatal^10^ COVID-19 due to an Omicron infection (**c**, **d**, respectively). **a** Includes 39,855 and 23,814 biologically independent samples for cases and controls, respectively, (**b**) includes 21,810 and 13,288 biologically independent samples for cases and controls, respectively, (**c**) includes 268 and 692 biologically independent samples for cases and controls, respectively, and (**d**) includes 164 and 404 biologically independent samples for cases and controls, respectively. Data are presented as effectiveness point estimates. Error bars indicate the corresponding 95% confidence intervals.

BNT162b2 effectiveness against any symptomatic Omicron infection, regardless of subvariant, was highest at 47.8% (95% CI: 40.8–53.9%) in the first 3 months after the second dose, but then declined to ~15% or below thereafter (Fig. 4a and Table 3). Effectiveness rapidly rebounded to 55.5% (95% CI: 49.3–61.0%) in the second and third weeks after the booster dose, but then gradually declined to 21.9% (95% CI: 7.7–33.9%) from the fourteenth week and thereafter. A similar pattern was observed for mRNA-1273 effectiveness (Fig. 4b and Table 4).

Effectiveness against any severe, critical, or fatal COVID-19 due to an Omicron infection, regardless of subvariant, was in the range of 70–80% at any time after the second dose for both the BNT162b2 and mRNA-1273 vaccines (Fig. 4c, d and Table 5). However, BNT162b2 effectiveness against any severe, critical, or fatal COVID-19 after the booster dose was greater than 90%. 95% CIs around estimates of mRNA-1273 effectiveness against any severe, critical, or fatal COVID-19 after the booster dose lacked adequate statistical precision—there were too few hospitalized COVID-19 cases among mRNA-1273 vaccinated persons (Table 5).

**Table 5:** Effectiveness of the BNT162b2 and mRNA-1273 vaccines against any severe^9^, critical^9^, or fatal^10^ COVID-19.

Sub-studies^a^	BNT162b2					mRNA-1273				
	Cases^b^ (Severe, critical, or fatal disease)^c^		Controls^b^ (PCR-negative)		Effectiveness in % (95% CI)^d^	Cases^b^ (Severe, critical, or fatal disease)^c^		Controls^b^ (PCR-negative)		Effectiveness in % (95% CI)^d^
	Vaccinated		Vaccinated			Vaccinated		Vaccinated		
	Yes	No	Yes	No		Yes	No	Yes	No	
Dose 1										
Dose 1 and no Dose 2	2	111	8	290	40.9 (−199.1 to 88.3)	0	110	3	287	100.0 (Omitted)^e^
Dose 2										
1–6 months after Dose 2 and no Dose 3	14	123	108	261	70.4 (45.0 to 84.0)	2	113	34	272	87.1 (40.2 to 97.2)
≥7 months after Dose 2 and no Dose 3	76	143	461	218	77.5 (67.8 to 84.3)	23	126	148	264	68.4 (46.1 to 81.5)
Dose 3 (booster dose)										
1–6 weeks after Dose 3	8	125	143	257	90.9 (78.6 to 96.1)	1	110	18	280	81.8 (−49.5 to 97.8)
≥7 weeks after Dose 3	12	134	197	254	90.1 (80.6 to 95.0)	0	110	3	287	100.0 (Omitted)^e^

*CI* confidence interval, *PCR* polymerase chain reaction.

^a^In each analysis for a specific time-since-vaccination stratum, we included only those vaccinated in this specific time-since-vaccination stratum and those unvaccinated. Only matched pairs of PCR-positive and PCR-negative persons, in which both members of the pair were either unvaccinated or fell within each time-since-vaccination stratum have been included in the corresponding vaccine effectiveness estimate. Thus, the number of cases (and controls) varied across time-since-vaccination analyses.

^b^Cases and controls were matched one-to-five by sex, 10-year age group, nationality, and calendar week of PCR test.

^c^Severity^9^, criticality^9^, and fatality^10^ were defined as per World Health Organization guidelines.

^d^Vaccine effectiveness was estimated using the test-negative, case–control study design^11,12^.

^e^Confidence interval could not be estimated using conditional logistic regression because of zero events among those vaccinated.

### Additional analyses

Sensitivity analyses adjusting for documented prior infection and healthcare worker status yielded similar findings to the main analyses (Supplementary Tables 1, 2). This is not unexpected as a strength of the test-negative design is its ability to disentangle one form of immunity from another, as validated through mathematical modeling analyses^20^. Sensitivity analyses to assess the impact of excluding children <12 years of age (Supplementary Tables 3, 4), or individuals <20 years of age (Supplementary Tables 5, 6), also yielded similar findings to the main analyses. A case-only analysis to examine differential waning for BA.1 versus BA.2 by comparing odds of BA.2 infection to odds of BA.1 infection among those vaccinated, with exposure being time-since vaccination, showed no evidence for a difference in the pattern of waning over time between the two subvariants (Supplementary Table 7).

## Discussion

No discernable differences were observed in the duration of mRNA vaccine protection against BA.1 versus BA.2 symptomatic infection. For each of these subvariants, vaccine effectiveness against symptomatic infection was ~50% in the first 3 months after the second dose, but declined to negligible levels thereafter. Effectiveness rapidly rebounded after the booster dose to reach similar levels to those seen right after the second dose, but waned again thereafter. There were also no discernable differences in effectiveness of BNT162b2 vaccine versus mRNA-1273 vaccine. Notably, the rapid waning in vaccine effectiveness against Omicron infections contrasts with the more durable protection for prior infection against Omicron reinfection^21,22^.

Despite only moderate and rapidly waning protection against symptomatic infection, mRNA vaccine effectiveness against COVID-19 hospitalization and death due to Omicron infections was strong at greater than 70% after the second dose. It was also higher after the booster dose at greater than 90%. These findings support the durability of vaccine protection against COVID-19 hospitalization and death for at least several months after receiving the second dose,^13,14,23^ but also demonstrate the importance of booster vaccination in achieving robust protection against any hospitalization and death due to Omicron infections. These findings suggest the need to consider rapid implementation of booster vaccination campaigns coincident with the emergence of a new wave or variant, at least to those most vulnerable to COVID-19 hospitalization and death.

This study has limitations. With the lower severity of Omicron infections^24,25^ and the young population of Qatar^15,26^, case numbers were insufficient to estimate the duration of protection against COVID-19 hospitalization and death for each subvariant separately. BA.1 and BA.2 ascertainment was based on proxy criteria, presence or absence of an S-gene “target failure” using the TaqPath PCR assay (Methods), but this method of ascertainment is well established not only for Omicron subvariants, but also for other variants such as Alpha^27–29^. Some Omicron infections may have been misclassified Delta infections, but this is not likely, as Delta incidence was limited during the study duration (Methods).

While nearly all third doses were administered as booster doses, few hundreds of them were administered as third primary-series doses for the immunocompromised population. However, this is not likely to affect our estimates as the number of immunocompromised individuals is very small in Qatar^30^, compared to the number of individuals who received the third dose as a booster dose. Vaccine protection was assessed for only several months after the second dose, and only several weeks after the booster dose. Longer-term protection against symptomatic infection and COVID-19 hospitalization and death remain uncertain. Vaccine effectiveness reached small but statistically significant negative values at 7 months or more after the second dose. Negative estimated effectiveness likely reflects an effect of bias and not true negative biological effectiveness. This bias may have risen from vaccinated persons having a higher social contact rate or adhering less to safety measures than unvaccinated persons^31–33^. With the high vaccine coverage among adults in Qatar (>85%)^13^, this bias may have also risen because the reference group of unvaccinated individuals included mainly children or young persons; therefore, it may not be representative of the wider population. However, sensitivity analyses excluding children and young persons confirmed the same study findings (Supplementary Tables 3–6). Unvaccinated adults are a small minority that may not be truly immune-naïve due to undocumented prior SARS-CoV-2 infections, especially now that we are two years into this pandemic. Earlier seroprevalence studies conducted in the same population have shown that a considerable proportion of infections went undocumented^17–19^. Bias due to depletion of the susceptible population may lead to underestimation of vaccine effectiveness^34^, even in the test-negative, case–control, study design, which is less prone to effect of this bias^13^.

While matching was done for sex, age, and nationality, this was not possible for other factors, such as comorbidities, as such data are not available. However, matching by these factors provided demonstrable control of bias in studies of different epidemiologic designs and that used control groups in Qatar^13,14,35–37^. Effectiveness was assessed using an observational, test-negative, case–control, study design^11,12^, rather than a design in which cohorts of vaccinated and unvaccinated individuals were followed up. However, the cohort study design applied earlier to the same population of Qatar yielded findings similar to those of the test-negative case–control design^36,38,39^, supporting the validity of this standard approach in assessing vaccine effectiveness^11,12,36,40^. Moreover, our recent study of the effectiveness of booster vaccination against any symptomatic Omicron infection, relative to that of the primary series, used a cohort study design^5^ and its results are consistent with results generated in the present study using the test-negative, case–control study design.

Nonetheless, one cannot exclude the possibility that in real-world data, bias could arise in unexpected ways, or from unknown sources, such as subtle differences in test-seeking behavior or changes in the pattern of testing with introduction of other testing modalities, such as rapid-antigen testing (RAT). For example, with the large Omicron wave in Qatar, use of RAT was expanded to supplement PCR testing starting from January 5, 2022. However, RAT was broadly implemented in the population and probably did not differentially affect PCR testing to introduce bias. With only 9% of Qatar’s population ≥50 years of age^15,41^, our findings may not be generalizable to other countries in which elderly citizens constitute a larger proportion of the total population.

Notwithstanding these limitations, consistent findings were reached, indicating rapid waning of vaccine protection against symptomatic Omicron infection that are consistent with findings of other studies for effectiveness against Omicron infection (with no BA.1/BA.2 subvariant specified)^42–48^. Moreover, with the mass scale of PCR testing in Qatar^13^, the likelihood of bias is perhaps minimized. Extensive sensitivity and additional analyses were conducted to investigate effects of potential bias in our earlier studies for the BNT162b2^13^ and mRNA-1273^14^ vaccines, which used the same methodology used here. These included different adjustments in the analysis, different approaches for factoring prior infection in the analysis, and different study inclusion and exclusion criteria to investigate whether effectiveness estimates could have been biased^13,14^. These analyses showed consistent findings^13,14^.

In conclusion, mRNA vaccines provide only moderate protection against symptomatic BA.1 and BA.2 Omicron infections, with no discernable differences in protection against either BA.1 or BA.2. Protection also wanes rapidly to negligible levels, starting 4 months after the second dose. Vaccine protection rebounds after booster vaccination, but also wanes thereafter. Meanwhile, vaccine protection against COVID-19 hospitalization and death is strong and durable after the second dose, and is most robust after a booster dose.

## Methods

### Oversight

Hamad Medical Corporation and Weill Cornell Medicine—Qatar Institutional Review Boards approved this retrospective study with waiver of informed consent. The study was reported following the Strengthening the Reporting of Observational Studies in Epidemiology (STROBE) guidelines. The STROBE checklist is found in Supplementary Table 8.

### Study population and data sources

This study was conducted in the resident population of Qatar, applying methodology that was developed earlier to assess duration of protection of the BNT162b2^13^ and mRNA-1273^14^ coronavirus disease 2019 (COVID-19) vaccines in the same population during earlier acute respiratory syndrome coronavirus 2 (SARS-CoV-2) infection waves. COVID-19 laboratory testing, vaccination, clinical infection data, and demographic details were extracted from the national, federated SARS-CoV-2 databases that include, with no missing information, all polymerase chain reaction (PCR) testing, COVID-19 vaccinations, and COVID-19 hospitalizations and deaths in Qatar since the start of the pandemic.

Every PCR test conducted in Qatar is categorized based on symptoms and the reason for testing. Qatar has young, international demographics. Only 9% of Qatar’s population is ≥50 years of age and 89% are international expatriates from over 150 countries^15,41^. The vast majority of individuals were vaccinated in Qatar, but if vaccinated elsewhere, those vaccinations were still registered in the health system at the port of entry upon arrival in Qatar.

### Study design

Vaccine effectiveness against symptomatic SARS-CoV-2 Omicron (B.1.1.529)^1^ infection during the large Omicron wave in Qatar, between December 23, 2021 and February 28, 2022, was estimated using the test-negative, case–control study design, a standard design for assessing vaccine effectiveness^11,12,36,40^. A symptomatic Omicron infection was defined as a nasopharyngeal PCR-positive swab collected during the Omicron wave because of clinical suspicion of infection, i.e., symptoms indicative of a respiratory tract infection. Cases (BA.1, BA.2, or any-Omicron-subvariant infected persons) and controls (uninfected persons) were exact-matched by sex, 10-year age group, nationality, and calendar week of PCR test. The ratio of matching in each analysis was determined based on available cases and controls (Fig. 2). Matching was implemented to control for established differences in the risk of exposure to SARS-CoV-2 infection in Qatar^15–19^.

Only the first PCR-positive test during the study was included for each case, whereas all PCR-negative tests during the study were included for each control. Controls included individuals with no record of a positive PCR or rapid-antigen test (RAT) during the study period. Only PCR tests conducted because of clinical suspicion of infection, i.e., symptoms indicative of a respiratory tract infection, were included in the analysis for cases and controls. All persons who received a vaccine other than BNT162b2 or mRNA-1273, or who received a different mix of vaccines, were excluded. These inclusion and exclusion criteria were implemented to minimize different types of potential bias based on earlier analyses in the same population^13,14^. Every case (or control) that met the inclusion criteria and that could be matched to a control (case) was included in the analysis. COVID-19 vaccination status was ascertained at the time of the PCR test. The age range for those with two and three BNT162b2 vaccine doses was 12–100 years and 13–97 years, respectively, among cases and 12–95 years and 12–97 years, respectively, among controls. The age range for those with two and three mRNA-1273 vaccine doses was 17–101 years and 20–81 years, respectively, among cases and 17–94 years and 18–92 years, respectively, among controls.

Vaccine effectiveness was also estimated against any severe, critical, or fatal COVID-19 infection due to Omicron, using the same methodology. Classification of COVID-19 case severity (acute-care hospitalizations)^9^, criticality (intensive-care-unit hospitalizations)^9^, and fatality^10^ followed World Health Organization (WHO) guidelines, and assessments were made by trained medical personnel using individual chart reviews (detailed description below). Each person who had a PCR-positive test result and COVID-19 hospital admission was subject to an infection severity assessment every three days until discharge or death, regardless of the hospital stay length or the time between the PCR-positive test and the final disease outcome. Individuals who progressed to severe^9^, critical^9^, or fatal^10^ COVID-19 between the PCR-positive test result and the end of the study were classified based on their worst outcome, starting with death, followed by critical disease, and then severe disease.

### COVID-19 severity, criticality, and fatality classification

WHO defines severe COVID-19 as a SARS-CoV-2 infected individual with “oxygen saturation of <90% on room air, and/or respiratory rate of >30 breaths/min in adults and children >5 years old (or ≥60 breaths/min in children <2 months old or ≥50 breaths/min in children 2–11 months old or ≥40 breaths/min in children 1–5 years old), and/or signs of severe respiratory distress (accessory muscle use and inability to complete full sentences, and, in children, very severe chest wall indrawing, grunting, central cyanosis, or presence of any other general danger signs)”^9^. Detailed criteria are in the WHO technical report^9^.

Critical COVID-19 is defined as a SARS-CoV-2 infected individual with “acute respiratory distress syndrome, sepsis, septic shock, or other conditions that would normally require the provision of life sustaining therapies such as mechanical ventilation (invasive or non-invasive) or vasopressor therapy”^9^. Detailed criteria are in the WHO technical report^9^.

COVID-19 death is defined as “a death resulting from a clinically compatible illness, in a probable or confirmed COVID-19 case, unless there is a clear alternative cause of death that cannot be related to COVID-19 disease (e.g., trauma). There should be no period of complete recovery from COVID-19 between illness and death. A death due to COVID-19 may not be attributed to another disease (e.g., cancer) and should be counted independently of preexisting conditions that are suspected of triggering a severe course of COVID-19”. Detailed criteria are in the WHO technical report^10^.

### Laboratory methods and subvariant ascertainment

#### Real-time reverse-transcription polymerase chain reaction testing

Nasopharyngeal and/or oropharyngeal swabs were collected for PCR testing and placed in Universal Transport Medium (UTM). Aliquots of UTM were: 1) extracted on KingFisher Flex (Thermo Fisher Scientific, USA), MGISP-960 (MGI, China), or ExiPrep 96 Lite (Bioneer, South Korea) followed by testing with real-time reverse-transcription PCR (RT-qPCR) using TaqPath COVID-19 Combo Kits (Thermo Fisher Scientific, USA) on an ABI 7500 FAST (Thermo Fisher Scientific, USA); 2) tested directly on the Cepheid GeneXpert system using the Xpert Xpress SARS-CoV-2 (Cepheid, USA); or 3) loaded directly into a Roche cobas 6800 system and assayed with the cobas SARS-CoV-2 Test (Roche, Switzerland). The first assay targets the viral S, N, and ORF1ab gene regions. The second targets the viral N and E-gene regions, and the third targets the ORF1ab and E-gene regions.

All PCR testing was conducted at the Hamad Medical Corporation Central Laboratory or at the Sidra Medicine Laboratory, following standardized protocols.

#### Classification of infections by subvariant

Surveillance for SARS-CoV-2 variants in Qatar is mainly based on viral genome sequencing and multiplex RT-qPCR variant screening^49^ of random positive clinical samples^2,13,36,38,50,51^, complemented by deep sequencing of wastewater samples^2,52^.

A total of 315 random SARS-CoV-2-positive specimens collected between December 19, 2021 and January 22, 2022 were viral whole-genome sequenced on a Nanopore GridION sequencing device. Of these, 300 (95.2%) were confirmed as Omicron infections and 15 (4.8%) as Delta (B.1.617.2)^1^ infections^2,4,5^. Of 286 Omicron infections with confirmed subvariant status, 68 (23.8%) were BA.1 cases and 218 (76.2%) were BA.2 cases.

Additionally, a total of 8811 random SARS-CoV-2-positive specimens collected between December 22, 2021 and February 28, 2022 were RT-qPCR genotyped. The RT-qPCR genotyping identified 470 B.1.617.2-like Delta case, 1017 BA.1-like Omicron cases, 4429 BA.2-like Omicron cases, and 2895 were undetermined cases where the genotype could not be assigned due to weak PCR Ct values.

The accuracy of the RT-qPCR genotyping was verified against either Sanger sequencing of the receptor-binding domain (RBD) of SARS-CoV-2 surface glycoprotein (S) gene, or by viral whole-genome sequencing on a Nanopore GridION sequencing device. From 147 random SARS-CoV-2-positive specimens all collected in December of 2021, RT-qPCR genotyping was able to assign a genotype in 129 samples. The agreement between RT-qPCR genotyping and sequencing was 100% for Delta (*n* = 82), 100% for Omicron BA.1 (*n* = 18), and 100% for Omicron BA.2 (*n* = 29). Of the remaining 18 specimens: 10 failed PCR amplification and sequencing, 8 could not be assigned a genotype by RT-qPCR (4 of 8 were B.1.617.2 by sequencing, and the remaining 4 failed sequencing). All the variant RT-qPCR genotyping was conducted at the Sidra Medicine Laboratory following standardized protocols.

The large Omicron-wave exponential-growth phase in Qatar started on December 19, 2021 and peaked in mid-January, 2022^2–5^. The study duration coincided with the intense Omicron wave where Delta incidence was limited. Accordingly, any PCR-positive test during the study duration, between December 23, 2021 and February 28, 2022, was assumed to be an Omicron infection. Of note that the study duration started on December 23, 2021, and not on December 19, 2021, to minimize the occurrence of residual Delta incidence during the first few days of the Omicron wave.

Informed by the viral genome sequencing and the RT-qPCR genotyping, a SARS-CoV-2 infection with the BA.1 subvariant was proxied as an S-gene “target failure” (SGTF) case using the TaqPath COVID-19 Combo Kit (Thermo Fisher Scientific, USA^53^) that tests for the S-gene and is affected by the del69/70 mutation in the S-gene^27^. A SARS-CoV-2 infection with the BA.2 subvariant was proxied as a non-SGTF case using this TaqPath Kit. While all PCR-confirmed infections were included in this study, subvariant status was only available for the 70.5% of PCR tests that were processed using the TaqPath Kit.

### Statistical analysis

Study samples were described using frequency distributions and measures of central tendency. Groups were compared using standardized mean differences (SMDs), defined as the difference in the mean of a covariate between groups, divided by the pooled standard deviation. SMD < 0.1 indicated adequate matching^54^. The odds ratio (and 95% confidence interval (CI)), comparing odds of vaccination among cases to that among controls, was estimated using conditional logistic regression factoring the matching in the study design. This analytical approach was implemented to reduce potential bias due to variation in epidemic phase^11,55^, gradual vaccination roll-out^11,55^, and other confounders^15,17–19,56,57^. CIs did not factor multiplicity and should not be used to infer definitive differences between study groups. Interactions were not examined. Vaccine effectiveness at different time frames and its associated 95% CI were then estimated using^11,12^:

$${{{{{\rm{Vaccine}}}}}}\,{{{{{\rm{effectiveness}}}}}}\,=\,1\,-\,{{{{{\rm{odds}}}}}}\,{{{{{\rm{ratio}}}}}}\,{{{{{\rm{of}}}}}}\,{{{{{\rm{vaccination}}}}}}\,{{{{{\rm{among}}}}}}\,{{{{{\rm{cases}}}}}}\,{{{{{\rm{versus}}}}}}\,{{{{{\rm{controls}}}}}}.$$

Since we used a test-negative study design, some persons were tested, PCR-positive or PCR-negative, after one vaccine dose, but before the next vaccine dose. This allowed us to estimate effectiveness after each dose. In each time-since-vaccination stratum, for first, second, and third doses, we analyzed only those vaccinated in this specific time-since-vaccination stratum and those unvaccinated (our reference group). Accordingly, the sample size for cases (and controls) varied in the different time-since-vaccination analyses. To assess booster effectiveness over longer time interval, the analysis for effectiveness against any symptomatic Omicron infection was subsequently extended until April 11, 2022. This extended analysis was done for only effectiveness against any symptomatic Omicron infection to optimize statistical precision with the larger case numbers. Effectiveness was estimated by one or more months in which 1 month was defined as 30 days, or by one or more weeks where 1 week was defined as 7 days.

Sensitivity analyses were conducted to assess the impact on effectiveness estimates of adjusting for documented prior infection and healthcare worker status in conditional logistic regression. With the majority of those unvaccinated being children or young persons, and therefore not necessarily representative of total population demographics, additional analyses were conducted to assess the impact of excluding children <12 years of age and individuals <20 years of age on effectiveness estimates. A case-only logistic regression analysis was conducted to examine differential waning for BA.1 versus BA.2, by comparing odds of BA.2 infection to odds of BA.1 infection among those vaccinated, with exposure being time-since vaccination, and with adjustment for sex, 10-year age groups, and 10 nationality groups. Statistical analyses were conducted in STATA/SE version 17.0^58^.

### Reporting summary

Further information on research design is available in the Nature Research Reporting Summary linked to this article.

## Supplementary information

Supplementary Information

Reporting Summary

## Data Availability

The dataset of this study is a property of the Qatar Ministry of Public Health that was provided to the researchers through a restricted-access agreement that prevents sharing the dataset with a third party or publicly. The data are available under restricted access for preservation of confidentiality of patient data. Access can be obtained through a direct application for data access to Her Excellency the Minister of Public Health (https://www.moph.gov.qa/english/OurServices/eservices/Pages/Governmental-Health-Communication-Center.aspx). The raw data are protected and are not available due to data privacy laws. Data were available to authors through.csv files where information has been downloaded from the CERNER database system (no links/accession codes were available to authors). Aggregate data are available within the paper and its Supplementary Information.

## Code availability

Standard epidemiological analyses were conducted using standard commands in STATA/SE 17.0^58^. The commands/code are accessible using URL: https://github.com/IDEGWCMQ/VE-Code/blob/main/VE%20Code.do^59^.
