# Supplementary material for: Duration of mRNA vaccine protection against SARS-CoV-2 Omicron BA.1 and BA.2 subvariants in Qatar
Source: Nat Commun. 2022 Jun 2;13:3082. doi: 10.1038/s41467-022-30895-3 (PMC9163167; doi:10.1038/s41467-022-30895-3)
Supplement: Supplementary file 1 — Supplementary Information [file 41467_2022_30895_MOESM1_ESM.pdf]

Supplementary Appendix

Table of contents

Supplementary Table 1. Sensitivity analysis for the effectiveness of the BNT162b2 vaccine against symptomatic SARS-CoV-2 BA.1 Omicron infection, BA.2 Omicron infection, and any Omicron infection*, adjusting for documented prior infection and health worker status in the conditional logistic regression. 2

Supplementary Table 2. Sensitivity analysis for the effectiveness of the mRNA-1273 vaccine against symptomatic SARS-CoV-2 BA.1 Omicron infection, BA.2 Omicron infection, and any Omicron infection*, adjusting for documented prior infection and health worker status in the conditional logistic regression. 3

Supplementary Table 3. Sensitivity analysis for the effectiveness of the BNT162b2 vaccine against symptomatic SARS-CoV-2 BA.1 Omicron infection, BA.2 Omicron infection, and any Omicron infection*, after excluding children <12 years of age. 4

Supplementary Table 4. Sensitivity analysis for the effectiveness of the mRNA-1273 vaccine against symptomatic SARS-CoV-2 BA.1 Omicron infection, BA.2 Omicron infection, and any Omicron infection*, after excluding children <12 years of age. 5

Supplementary Table 5. Sensitivity analysis for the effectiveness of the BNT162b2 vaccine against symptomatic SARS-CoV-2 BA.1 Omicron infection, BA.2 Omicron infection, and any Omicron infection*, after excluding individuals <20 years of age. 6

Supplementary Table 6. Sensitivity analysis for the effectiveness of the mRNA-1273 vaccine against symptomatic SARS-CoV-2 BA.1 Omicron infection, BA.2 Omicron infection, and any Omicron infection*, after excluding individuals <20 years of age. 7

Supplementary Table 7. Case-only analysis to examine differential waning for BA.1 versus BA.2 by comparing odds of BA.2 infection to odds of BA.1 infection among those vaccinated, with exposure being time since vaccination. 8

Supplementary Table 8. STROBE checklist for case-control studies. 9

References 11

Supplementary Table 1. Sensitivity analysis for the effectiveness of the BNT162b2 vaccine against symptomatic SARS-CoV-2 BA.1 Omicron infection, BA.2 Omicron infection, and any Omicron infection*, adjusting for documented prior infection and health worker status in the conditional logistic regression.

Sub-studies†	Cases (PCR-positive)		Controls (PCR-negative)		Effectiveness in % (95% CI)‡
	Vaccinated	Unvaccinated	Vaccinated	Unvaccinated	
**Effectiveness against symptomatic BA.1 Omicron infection§**					
**Dose 1**					
0-13 days after Dose 1 and no Dose 2	10	1,969	20	3,456	6.2 (-115.9 to 59.3)
≥14 days after Dose 1 and no Dose 2	25	1,968	66	3,441	35.2 (-6.0 to 60.3)
**Dose 2**					
1-3 months after Dose 2 and no Dose 3	130	1,992	376	3,409	43.4 (28.8 to 55.1)
4-6 months after Dose 2 and no Dose 3	502	2,004	941	3,506	3.6 (-10.6 to 15.9)
≥7 months after Dose 2 and no Dose 3	3,570	2,060	6,007	3,947	-17.8 (-28.8 to -7.8)
**Dose 3 (booster dose)**					
<1 month after Dose 3	180	2,008	622	3,339	58.3 (48.7 to 66.2)
≥1 month after Dose 3	483	2,031	1,145	3,441	41.6 (31.0 to 50.6)
**Effectiveness against symptomatic BA.2 Omicron infection¶**					
**Dose 1**					
0-13 days after Dose 1 and no Dose 2	32	5,744	34	5,742	-2.1 (-67.3 to 37.7)
≥14 days after Dose 1 and no Dose 2	64	5,774	99	5,739	35.2 (10.1 to 53.4)
**Dose 2**					
1-3 months after Dose 2 and no Dose 3	263	5,964	496	5,731	48.3 (39.0 to 56.2)
4-6 months after Dose 2 and no Dose 3	1,203	5,924	1,318	5,809	9.1 (-0.1 to 17.5)
≥7 months after Dose 2 and no Dose 3	8,003	5,840	7,762	6,081	-13.7 (-21.0 to -6.8)
**Dose 3 (booster dose)**					
<1 month after Dose 3	709	6,038	1,034	5,713	44.6 (37.2 to 51.2)
≥1 month after Dose 3	1,580	6,211	2,029	5,762	42.0 (35.6 to 47.8)
**Effectiveness against any symptomatic Omicron infection****					
**Dose 1**					
0-13 days after Dose 1 and no Dose 2	56	12,174	34	7,278	5.9 (-47.1 to 39.8)
≥14 days after Dose 1 and no Dose 2	151	12,205	130	7,276	29.6 (9.6 to 45.2)
**Dose 2**					
1-3 months after Dose 2 and no Dose 3	585	12,623	599	7,309	44.8 (37.2 to 51.5)
4-6 months after Dose 2 and no Dose 3	2,479	12,590	1,605	7,333	12.1 (5.0 to 18.7)
≥7 months after Dose 2 and no Dose 3	16,435	12,564	9,073	7,637	-9.5 (-15.2 to -4.1)
**Dose 3 (booster dose)**					
<1 month after Dose 3	1,326	12,777	1,300	7,275	49.9 (44.5 to 54.8)
≥1 month after Dose 3	3,120	13,040	2,484	7,360	42.9 (37.7 to 47.6)

Abbreviations: CI, confidence interval; PCR, polymerase chain reaction.

*A symptomatic infection was defined as a PCR-positive nasopharyngeal swab conducted because of clinical suspicion due to presence of symptoms compatible with a respiratory tract infection.

†In each analysis for a specific time-since-vaccination stratum, we included only those vaccinated in this specific time-since-vaccination stratum and those unvaccinated. Only matched pairs of PCR-positive and PCR-negative persons, in which both members of the pair were either unvaccinated or fell within each time-since-vaccination stratum have been included in the corresponding vaccine effectiveness estimate. Thus, the number of cases (and controls) varied across time-since-vaccination analyses.

‡Vaccine effectiveness was estimated using the test-negative, case-control study design[1, 2].

§Cases and controls were matched one-to-two by sex, 10-year age group, nationality, and calendar week of PCR test.

¶Cases and controls were matched one-to-one by sex, 10-year age group, nationality, and calendar week of PCR test.

**Cases and controls were matched two-to-one by sex, 10-year age group, nationality, and calendar week of PCR test.

Supplementary Table 2. Sensitivity analysis for the effectiveness of the mRNA-1273 vaccine against symptomatic SARS-CoV-2 BA.1 Omicron infection, BA.2 Omicron infection, and any Omicron infection*, adjusting for documented prior infection and health worker status in the conditional logistic regression.

Sub-studies†	Cases (PCR-positive)		Controls (PCR-negative)		Effectiveness in % (95% CI)‡
	Vaccinated	Unvaccinated	Vaccinated	Unvaccinated	
**Effectiveness against symptomatic BA.1 Omicron infection§**					
**Dose 1**					
0-13 days after Dose 1 and no Dose 2	3	1,942	8	3,400	45.8 (-115.2 to 86.3)
≥14 days after Dose 1 and no Dose 2	14	1,942	19	3,405	-40.1 (-191.7 to 32.7)
**Dose 2**					
1-3 months after Dose 2 and no Dose 3	6	1,943	27	3,396	71.0 (22.6 to 89.1)
4-6 months after Dose 2 and no Dose 3	289	1,976	667	3,377	27.1 (13.6 to 38.5)
≥7 months after Dose 2 and no Dose 3	1,125	1,999	1,847	3,638	-16.8 (-31.1 to -4.1)
**Dose 3 (booster dose)**					
<1 month after Dose 3	55	1,951	182	3,377	48.9 (27.9 to 63.8)
≥1 month after Dose 3	36	1,953	102	3,396	46.0 (17.2 to 64.8)
**Effectiveness against symptomatic BA.2 Omicron infection¶**					
**Dose 1**					
0-13 days after Dose 1 and no Dose 2	8	5,651	10	5,649	3.0 (-151.5 to 62.6)
≥14 days after Dose 1 and no Dose 2	31	5,645	27	5,649	-19.9 (-104.9 to 29.9)
**Dose 2**					
1-3 months after Dose 2 and no Dose 3	26	5,664	40	5,650	39.7 (-1.2 to 64.1)
4-6 months after Dose 2 and no Dose 3	989	5,756	1,059	5,686	6.0 (-4.9 to 15.8)
≥7 months after Dose 2 and no Dose 3	2,917	5,627	2,686	5,858	-24.0 (-34.5 to -14.3)
**Dose 3 (booster dose)**					
<1 month after Dose 3	164	5,727	250	5,641	36.7 (20.9 to 49.2)
≥1 month after Dose 3	92	5,709	149	5,652	42.5 (23.6 to 56.7)
**Effectiveness against any symptomatic Omicron infection****					
**Dose 1**					
0-13 days after Dose 1	17	11,987	11	7,153	3.7 (-108.9 to 55.6)
≥14 days after Dose 1 and no Dose 2	52	11,984	36	7,150	5.8 (-47.0 to 39.7)
**Dose 2**					
1-3 months after Dose 2 and no Dose 3	47	12,014	51	7,151	39.7 (8.8 to 60.2)
4-6 months after Dose 2 and no Dose 3	1,863	12,321	1,294	7,205	15.0 (7.0 to 22.3)
≥7 months after Dose 2 and no Dose 3	5,820	12,144	3,112	7,374	-17.0 (-25.0 to -9.5)
**Dose 3 (booster dose)**					
<1 month after Dose 3	323	12,156	321	7,148	40.8 (29.6 to 50.3)
≥1 month after Dose 3	169	12,100	181	7,155	48.3 (34.7 to 59.1)

Abbreviations: CI, confidence interval; PCR, polymerase chain reaction.

*A symptomatic infection was defined as a PCR-positive nasopharyngeal swab conducted because of clinical suspicion due to presence of symptoms compatible with a respiratory tract infection.

†In each analysis for a specific time-since-vaccination stratum, we included only those vaccinated in this specific time-since-vaccination stratum and those unvaccinated. Only matched pairs of PCR-positive and PCR-negative persons, in which both members of the pair were either unvaccinated or fell within each time-since-vaccination stratum have been included in the corresponding vaccine effectiveness estimate. Thus, the number of cases (and controls) varied across time-since-vaccination analyses.

‡Vaccine effectiveness was estimated using the test-negative, case-control study design[1, 2].

§Cases and controls were matched one-to-two by sex, 10-year age group, nationality, and calendar week of PCR test.

¶Cases and controls were matched one-to-one by sex, 10-year age group, nationality, and calendar week of PCR test.

**Cases and controls were matched two-to-one by sex, 10-year age group, nationality, and calendar week of PCR test.

Supplementary Table 3. Sensitivity analysis for the effectiveness of the BNT162b2 vaccine against symptomatic SARS-CoV-2 BA.1 Omicron infection, BA.2 Omicron infection, and any Omicron infection*, after excluding children <12 years of age.

Sub-studies†	Cases (PCR-positive)		Controls (PCR-negative)		Effectiveness in % (95% CI)‡
	Vaccinated	Unvaccinated	Vaccinated	Unvaccinated	
**Effectiveness against symptomatic BA.1 Omicron infection§**					
**Dose 1**					
0-13 days after Dose 1 and no Dose 2	10	1,155	21	1,886	23.4 (-64.2 to 64.2)
≥14 days after Dose 1 and no Dose 2	25	1,155	65	1,874	37.6 (0.3 to 60.9)
**Dose 2**					
1-3 months after Dose 2 and no Dose 3	127	1,181	351	1,858	45.1 (31.1 to 56.3)
4-6 months after Dose 2 and no Dose 3	499	1,195	960	1,921	14.1 (1.3 to 25.2)
≥7 months after Dose 2 and no Dose 3	3,564	1,251	6,001	2,380	-17.3 (-28.0 to -7.5)
**Dose 3 (booster dose)**					
<1 month after Dose 3	181	1,192	637	1,754	61.0 (52.7 to 67.9)
≥1 month after Dose 3	483	1,218	1,171	1,847	42.4 (33.2 to 50.4)
**Effectiveness against symptomatic BA.2 Omicron infection¶**					
**Dose 1**					
0-13 days after Dose 1 and no Dose 2	26	2,973	30	2,969	13.3 (-46.5 to 48.7)
≥14 days after Dose 1 and no Dose 2	67	2,996	97	2,966	31.6 (6.2 to 50.1)
**Dose 2**					
1-3 months after Dose 2 and no Dose 3	257	3,199	488	2,968	51.4 (42.9 to 58.7)
4-6 months after Dose 2 and no Dose 3	1,233	3,121	1,319	3,035	10.0 (0.8 to 18.3)
≥7 months after Dose 2 and no Dose 3	8,001	3,064	7,742	3,323	-13.7 (-21.0 to -6.9)
**Dose 3 (booster dose)**					
<1 month after Dose 3	705	3,265	1,057	2,913	44.5 (37.6 to 50.6)
≥1 month after Dose 3	1,604	3,412	2,025	2,991	38.9 (32.7 to 44.5)
**Effectiveness against any symptomatic Omicron infection****					
**Dose 1**					
0-13 days after Dose 1 and no Dose 2	55	6,043	32	3,619	3.4 (-51.0 to 38.2)
≥14 days after Dose 1 and no Dose 2	133	6,097	128	3,616	37.2 (19.4 to 51.1)
**Dose 2**					
1-3 months after Dose 2 and no Dose 3	616	6,466	588	3,656	43.8 (36.3 to 50.5)
4-6 months after Dose 2 and no Dose 3	2,511	6,413	1,586	3,689	13.1 (6.0 to 19.7)
≥7 months after Dose 2 and no Dose 3	16,552	6,302	9,025	4,014	-15.0 (-21.0 to -9.4)
**Dose 3 (booster dose)**					
<1 month after Dose 3	1,323	6,648	1,278	3,634	48.0 (42.7 to 52.8)
≥1 month after Dose 3	3,113	6,919	2,494	3,689	40.7 (35.8 to 45.3)

Abbreviations: CI, confidence interval; PCR, polymerase chain reaction.

*A symptomatic infection was defined as a PCR-positive nasopharyngeal swab conducted because of clinical suspicion due to presence of symptoms compatible with a respiratory tract infection.

†In each analysis for a specific time-since-vaccination stratum, we included only those vaccinated in this specific time-since-vaccination stratum and those unvaccinated. Only matched pairs of PCR-positive and PCR-negative persons, in which both members of the pair were either unvaccinated or fell within each time-since-vaccination stratum have been included in the corresponding vaccine effectiveness estimate. Thus, the number of cases (and controls) varied across time-since-vaccination analyses.

‡Vaccine effectiveness was estimated using the test-negative, case-control study design[1, 2].

§Cases and controls were matched one-to-two by sex, 10-year age group, nationality, and calendar week of PCR test.

¶Cases and controls were matched one-to-one by sex, 10-year age group, nationality, and calendar week of PCR test.

**Cases and controls were matched two-to-one by sex, 10-year age group, nationality, and calendar week of PCR test.

Supplementary Table 4. Sensitivity analysis for the effectiveness of the mRNA-1273 vaccine against symptomatic SARS-CoV-2 BA.1 Omicron infection, BA.2 Omicron infection, and any Omicron infection*, after excluding children <12 years of age.

Sub-studies†	Cases (PCR-positive)		Controls (PCR-negative)		Effectiveness in % (95% CI)‡
	Vaccinated	Unvaccinated	Vaccinated	Unvaccinated	
**Effectiveness against symptomatic BA.1 Omicron infection§**					
**Dose 1**					
0-13 days after Dose 1 and no Dose 2	3	1,128	9	1,830	48.5 (-93.2 to 86.2)
≥14 days after Dose 1 and no Dose 2	14	1,128	17	1,838	-29.5 (-168.7 to 37.6)
**Dose 2**					
1-3 months after Dose 2 and no Dose 3	6	1,129	30	1,824	70.1 (27.2 to 87.7)
4-6 months after Dose 2 and no Dose 3	283	1,169	664	1,807	33.7 (21.6 to 44.0)
≥7 months after Dose 2 and no Dose 3	1,125	1,184	1,830	2,074	-10.6 (-23.6 to 1.0)
**Dose 3 (booster dose)**					
<1 month after Dose 3	54	1,139	184	1,807	52.0 (33.4 to 65.4)
≥1 month after Dose 3	36	1,139	101	1,827	43.4 (15.5 to 62.1)
**Effectiveness against symptomatic BA.2 Omicron infection¶**					
**Dose 1**					
0-13 days after Dose 1 and no Dose 2	8	2,876	9	2,875	11.1 (-130.3 to 65.7)
≥14 days after Dose 1 and no Dose 2	22	2,879	29	2,872	25.0 (-32.1 to 57.4)
**Dose 2**					
1-3 months after Dose 2 and no Dose 3	18	2,897	37	2,878	51.4 (14.6 to 72.3)
4-6 months after Dose 2 and no Dose 3	945	3,022	1,059	2,908	16.1 (6.4 to 24.7)
≥7 months after Dose 2 and no Dose 3	2,861	2,906	2,676	3,091	-16.0 (-25.6 to -7.3)
**Dose 3 (booster dose)**					
<1 month after Dose 3	169	2,947	265	2,851	40.7 (26.9 to 51.9)
≥1 month after Dose 3	84	2,941	153	2,872	47.9 (31.1 to 60.6)
**Effectiveness against any symptomatic Omicron infection****					
**Dose 1**					
0-13 days after Dose 1 and no Dose 2	19	5,856	9	3,495	-17.6 (-161.4 to 47.1)
≥14 days after Dose 1 and no Dose 2	40	5,868	36	3,490	28.9 (-12.3 to 54.9)
**Dose 2**					
1-3 months after Dose 2 and no Dose 3	43	5,890	46	3,497	45.2 (16.0 to 64.2)
4-6 months after Dose 2 and no Dose 3	1,878	6,175	1,293	3,544	18.2 (10.6 to 25.1)
≥7 months after Dose 2 and no Dose 3	5,832	6,001	3,107	3,716	-15.0 (-22.7 to -7.8)
**Dose 3 (booster dose)**					
<1 month after Dose 3	327	6,027	320	3,490	44.4 (33.8 to 53.2)
≥1 month after Dose 3	174	5,965	179	3,498	46.0 (32.4 to 56.9)

Abbreviations: CI, confidence interval; PCR, polymerase chain reaction.

*A symptomatic infection was defined as a PCR-positive nasopharyngeal swab conducted because of clinical suspicion due to presence of symptoms compatible with a respiratory tract infection.

†In each analysis for a specific time-since-vaccination stratum, we included only those vaccinated in this specific time-since-vaccination stratum and those unvaccinated. Only matched pairs of PCR-positive and PCR-negative persons, in which both members of the pair were either unvaccinated or fell within each time-since-vaccination stratum have been included in the corresponding vaccine effectiveness estimate. Thus, the number of cases (and controls) varied across time-since-vaccination analyses.

‡Vaccine effectiveness was estimated using the test-negative, case-control study design[1, 2].

§Cases and controls were matched one-to-two by sex, 10-year age group, nationality, and calendar week of PCR test.

¶Cases and controls were matched one-to-one by sex, 10-year age group, nationality, and calendar week of PCR test.

**Cases and controls were matched two-to-one by sex, 10-year age group, nationality, and calendar week of PCR test.

Supplementary Table 5. Sensitivity analysis for the effectiveness of the BNT162b2 vaccine against symptomatic SARS-CoV-2 BA.1 Omicron infection, BA.2 Omicron infection, and any Omicron infection*, after excluding individuals <20 years of age.

Sub-studies†	Cases (PCR-positive)		Controls (PCR-negative)		Effectiveness in % (95% CI)‡
	Vaccinated	Unvaccinated	Vaccinated	Unvaccinated	
**Effectiveness against symptomatic BA.1 Omicron infection§**					
**Dose 1**					
0-13 days after Dose 1 and no Dose 2	9	1,034	19	1,677	24.9 (-71.7 to 67.2)
≥14 days after Dose 1 and no Dose 2	18	1,035	56	1,669	47.6 (10.0 to 69.5)
**Dose 2**					
1-3 months after Dose 2 and no Dose 3	87	1,050	259	1,648	48.1 (32.4 to 60.1)
4-6 months after Dose 2 and no Dose 3	348	1,055	628	1,710	9.4 (-6.1 to 22.7)
≥7 months after Dose 2 and no Dose 3	3,203	1,115	5,357	2,130	-18.4 (-29.9 to -7.9)
**Dose 3 (booster dose)**					
<1 month after Dose 3	177	1,070	607	1,570	60.8 (52.0 to 68.0)
≥1 month after Dose 3	480	1,096	1,141	1,661	40.0 (30.4 to 48.4)
**Effectiveness against symptomatic BA.2 Omicron infection¶**					
**Dose 1**					
0-13 days after Dose 1 and no Dose 2	26	2,674	26	2,674	0.0 (-72.2 to 41.9)
≥14 days after Dose 1 and no Dose 2	58	2,699	88	2,669	35.7 (9.5 to 54.3)
**Dose 2**					
1-3 months after Dose 2 and no Dose 3	196	2,826	352	2,670	48.0 (37.4 to 56.8)
4-6 months after Dose 2 and no Dose 3	955	2,701	908	2,748	-7.5 (-19.9 to 3.6)
≥7 months after Dose 2 and no Dose 3	7,404	2,605	6,986	3,023	-27.0 (-35.7 to -18.8)
**Dose 3 (booster dose)**					
<1 month after Dose 3	724	2,937	1,028	2,633	41.5 (34.1 to 48.1)
≥1 month after Dose 3	1,612	3,096	2,003	2,705	36.6 (30.2 to 42.4)
**Effectiveness against any symptomatic Omicron infection****					
**Dose 1**					
0-13 days after Dose 1 and no Dose 2	42	5,434	27	3,261	12.9 (-42.3 to 46.7)
≥14 days after Dose 1 and no Dose 2	107	5,484	107	3,261	38.7 (19.3 to 53.5)
**Dose 2**					
1-3 months after Dose 2 and no Dose 3	458	5,713	416	3,291	39.5 (30.0 to 47.7)
4-6 months after Dose 2 and no Dose 3	1,860	5,561	1,065	3,317	-0.04 (-9.5 to 8.6)
≥7 months after Dose 2 and no Dose 3	15,089	5,543	8,167	3,610	-19.6 (-26.2 to -13.3)
**Dose 3 (booster dose)**					
<1 month after Dose 3	1,316	6,012	1,270	3,266	47.4 (42.0 to 52.3)
≥1 month after Dose 3	3,107	6,283	2,458	3,349	38.9 (33.9 to 43.6)

Abbreviations: CI, confidence interval; PCR, polymerase chain reaction.

*A symptomatic infection was defined as a PCR-positive nasopharyngeal swab conducted because of clinical suspicion due to presence of symptoms compatible with a respiratory tract infection.

†In each analysis for a specific time-since-vaccination stratum, we included only those vaccinated in this specific time-since-vaccination stratum and those unvaccinated. Only matched pairs of PCR-positive and PCR-negative persons, in which both members of the pair were either unvaccinated or fell within each time-since-vaccination stratum have been included in the corresponding vaccine effectiveness estimate. Thus, the number of cases (and controls) varied across time-since-vaccination analyses.

‡Vaccine effectiveness was estimated using the test-negative, case-control study design[1, 2].

§Cases and controls were matched one-to-two by sex, 10-year age group, nationality, and calendar week of PCR test.

¶Cases and controls were matched one-to-one by sex, 10-year age group, nationality, and calendar week of PCR test.

**Cases and controls were matched two-to-one by sex, 10-year age group, nationality, and calendar week of PCR test.

Supplementary Table 6. Sensitivity analysis for the effectiveness of the mRNA-1273 vaccine against symptomatic SARS-CoV-2 BA.1 Omicron infection, BA.2 Omicron infection, and any Omicron infection*, after excluding individuals <20 years of age.

Sub-studies†	Cases (PCR-positive)		Controls (PCR-negative)		Effectiveness in % (95% CI)‡
	Vaccinated	Unvaccinated	Vaccinated	Unvaccinated	
**Effectiveness against symptomatic BA.1 Omicron infection§**					
**Dose 1**					
0-13 days after Dose 1 and no Dose 2	3	1,011	7	1,630	40.8 (-132.7 to 85.0)
≥14 days after Dose 1 and no Dose 2	14	1,011	17	1,637	-28.3 (-162.9 to 37.4)
**Dose 2**					
1-3 months after Dose 2 and no Dose 3	6	1,012	27	1,625	65.1 (14.9 to 85.7)
4-6 months after Dose 2 and no Dose 3	282	1,043	673	1,580	34.6 (22.8 to 44.6)
≥7 months after Dose 2 and no Dose 3	1,106	1,070	1,807	1,873	-9.7 (-22.8 to 1.9)
**Dose 3 (booster dose)**					
<1 month after Dose 3	54	1,021	178	1,610	50.6 (31.1 to 64.6)
≥1 month after Dose 3	37	1,021	91	1,636	32.5 (-0.4 to 54.6)
**Effectiveness against symptomatic BA.2 Omicron infection¶**					
**Dose 1**					
0-13 days after Dose 1 and no Dose 2	8	2,593	7	2,594	-14.3 (-215.2 to 58.6)
≥14 days after Dose 1 and no Dose 2	28	2,591	27	2,592	-3.8 (-77.9 to 39.4)
**Dose 2**					
1-3 months after Dose 2 and no Dose 3	22	2,609	42	2,589	48.8 (13.3 to 69.7)
4-6 months after Dose 2 and no Dose 3	950	2,719	1,025	2,644	10.9 (0.7 to 20.1)
≥7 months after Dose 2 and no Dose 3	2,866	2,587	2,624	2,829	-21.9 (-32.0 to -12.6)
**Dose 3 (booster dose)**					
<1 month after Dose 3	167	2,665	255	2,577	37.9 (23.6 to 49.6)
≥1 month after Dose 3	93	2,648	150	2,591	41.6 (23.1 to 55.7)
**Effectiveness against any symptomatic Omicron infection****					
**Dose 1**					
0-13 days after Dose 1 and no Dose 2	17	5,266	8	3,151	-16.3 (-171.0 to 50.1)
≥14 days after Dose 1 and no Dose 2	47	5,271	31	3,151	7.2 (-47.3 to 41.5)
**Dose 2**					
1-3 months after Dose 2 and no Dose 3	46	5,294	47	3,150	42.8 (13.0 to 62.5)
4-6 months after Dose 2 and no Dose 3	1,877	5,551	1,270	3,203	16.0 (8.2 to 23.2)
≥7 months after Dose 2 and no Dose 3	5,763	5,402	3,076	3,357	-14.4 (-22.1 to -7.2)
**Dose 3 (booster dose)**					
<1 month after Dose 3	296	5,461	319	3,142	47.5 (37.7 to 55.8)
≥1 month after Dose 3	166	5,376	170	3,159	44.4 (30.2 to 55.8)

Abbreviations: CI, confidence interval; PCR, polymerase chain reaction.

*A symptomatic infection was defined as a PCR-positive nasopharyngeal swab conducted because of clinical suspicion due to presence of symptoms compatible with a respiratory tract infection.

†In each analysis for a specific time-since-vaccination stratum, we included only those vaccinated in this specific time-since-vaccination stratum and those unvaccinated. Only matched pairs of PCR-positive and PCR-negative persons, in which both members of the pair were either unvaccinated or fell within each time-since-vaccination stratum have been included in the corresponding vaccine effectiveness estimate. Thus, the number of cases (and controls) varied across time-since-vaccination analyses.

‡Vaccine effectiveness was estimated using the test-negative, case-control study design[1, 2].

§Cases and controls were matched one-to-two by sex, 10-year age group, nationality, and calendar week of PCR test.

¶Cases and controls were matched one-to-one by sex, 10-year age group, nationality, and calendar week of PCR test.

**Cases and controls were matched two-to-one by sex, 10-year age group, nationality, and calendar week of PCR test.

Supplementary Table 7. Case-only analysis to examine differential waning for BA.1 versus BA.2 by comparing odds of BA.2 infection to odds of BA.1 infection among those vaccinated, with exposure being time since vaccination.

Time since vaccination	BNT162b2 vaccine	mRNA-1273 vaccine
	AOR (95% CI)*	AOR (95% CI)*
**Dose 2**		
1-3 months after Dose 2 and no Dose 3	Reference	Reference
4-6 months after Dose 2 and no Dose 3	1.11 (0.90-1.37)	0.81 (0.38-1.74)
≥7 months after Dose 2 and no Dose 3	1.01 (0.83-1.23)	0.80 (0.38-1.70)
**Dose 3 (booster dose)**		
1 week after Dose 3	1.33 (0.96-1.84)	0.93 (0.37-2.32)
2-3 weeks after Dose 3	1.27 (0.94-1.70)	0.76 (0.33-1.78)
4-5 weeks after Dose 3	1.00 (0.76-1.32)	0.78 (0.32-1.87)
6-7 weeks after Dose 3	0.83 (0.65-1.07)	0.55 (0.24-1.28)
8-9 weeks after Dose 3	1.01 (0.76-1.34)	--
10-11 weeks after Dose 3	0.96 (0.68-1.34)	--
12-13 weeks after Dose 3	1.09 (0.75-1.57)	--
≥14 weeks after Dose 3	1.36 (0.98-1.88)	--

Abbreviations: AOR, adjusted odds ratio; CI, confidence interval.
*AORs were derived using logistic regression that compared odds of SARS-CoV-2 BA.2 Omicron infection to odds of SARS-CoV-2 BA.1 Omicron infection adjusting for sex, 10-year age groups, and 10 nationality groups.

Supplementary Table 8. STROBE checklist for case-control studies.

	Item No	Recommendation	Main text page
**Title and abstract**	1	(*a*) Indicate the study's design with a commonly used term in the title or the abstract	Abstract
		(*b*) Provide in the abstract an informative and balanced summary of what was done and what was found	Abstract
**Introduction**			
Background/rationale	2	Explain the scientific background and rationale for the investigation being reported	Introduction
Objectives	3	State specific objectives, including any prespecified hypotheses	Introduction
**Methods**			
Study design	4	Present key elements of study design	Methods ('Study design')
Setting	5	Describe the setting, locations, and relevant dates, including periods of recruitment, exposure, follow-up, and data collection	Methods ('Study design') & Figure 2
Participants	6	(*a*) Give the eligibility criteria, and the sources and methods of case ascertainment and control selection. Give the rationale for the choice of cases and controls	Methods ("Study population and data sources' & 'Study design') & Figure 2
		(*b*) For matched studies, give matching criteria and the number of controls per case	
Variables	7	Clearly define all outcomes, exposures, predictors, potential confounders, and effect modifiers. Give diagnostic criteria, if applicable	Methods ('Study design', 'COVID-19 severity, criticality, and fatality classification', & 'Laboratory methods and subvariant ascertainment'), Tables 1 & 2, & Figure2
Data sources/ measurement	8	For each variable of interest, give sources of data and details of methods of assessment (measurement). Describe comparability of assessment methods if there is more than one group	Methods, Tables 1 & 2, & Figure2
Bias	9	Describe any efforts to address potential sources of bias	Methods ('Study design' & 'Statistical analysis')
Study size	10	Explain how the study size was arrived at	Figure 2
Quantitative variables	11	Explain how quantitative variables were handled in the analyses. If applicable, describe which groupings were chosen and why	Methods ('Study design' & 'Statistical analysis') & Tables 1 & 2
Statistical methods	12	(*a*) Describe all statistical methods, including those used to control for confounding	Methods ('Statistical analysis)
		(*b*) Describe any methods used to examine subgroups and interactions	Methods ('Statistical analysis)
		(*c*) Explain how missing data were addressed	NA, see Methods ('Study population and data sources')
		(*d*) If applicable, explain how matching of cases and controls was addressed	Methods ('Study design'& 'Statistical analysis')
		(*e*) Describe any sensitivity analyses	Methods ('Statistical analysis')
**Results**			
Participants	13	(a) Report numbers of individuals at each stage of study—eg numbers potentially eligible, examined for eligibility, confirmed eligible, included in the study, completing follow-up, and analysed	Figure 2 & Tables 1 & 2
		(b) Give reasons for non-participation at each stage	
		(c) Consider use of a flow diagram	
Descriptive data	14	(a) Give characteristics of study participants (eg demographic, clinical, social) and information on exposures and potential confounders	Results ('Main analyses", paragraphs 1 & 2) & Tables 1-2
		(b) Indicate number of participants with missing data for each variable of interest	NA, see Methods ('Study population and data sources')
Outcome data	15	Report numbers in each exposure category, or summary measures of exposure	Results ('Main analyses", paragraphs 3-5), Figures 3 & 4, & Tables 3 & 4
Main results	16	(*a*) Give unadjusted estimates and, if applicable, confounder-adjusted estimates and their precision (eg, 95% confidence interval). Make clear which confounders were adjusted for and why they were included	Results ('Main analyses", paragraphs 3-5), Figures 3 & 4, & Tables 3 & 4
		(*b*) Report category boundaries when continuous variables were categorized	Tables 1 & 2
		(*c*) If relevant, consider translating estimates of relative risk into absolute risk for a meaningful time period	NA
Other analyses	17	Report other analyses done—eg analyses of subgroups and interactions, and sensitivity analyses	Results ('Additional analyses') & Supp. Tables 1-7
**Discussion**			
Key results	18	Summarise key results with reference to study objectives	Discussion, paragraphs 1 & 2

Limitations	19	Discuss limitations of the study, taking into account sources of potential bias or imprecision. Discuss both direction and magnitude of any potential bias	Discussion, paragraphs 3-6
Interpretation	20	Give a cautious overall interpretation of results considering objectives, limitations, multiplicity of analyses, results from similar studies, and other relevant evidence	Discussion, paragraphs 7 & 8
Generalisability	21	Discuss the generalisability (external validity) of the study results	Discussion, paragraph 6
**Other information**			
Funding	22	Give the source of funding and the role of the funders for the present study and, if applicable, for the original study on which the present article is based	Acknowledgements

Abbreviations: NA, not applicable; p. page; Supp. Supplementary.

References

1. Jackson ML, Nelson JC. The test-negative design for estimating influenza vaccine effectiveness. *Vaccine* **31**, 2165-2168 (2013).

2. Verani JR*, et al.* Case-control vaccine effectiveness studies: Preparation, design, and enrollment of cases and controls. *Vaccine* **35**, 3295-3302 (2017).
