# Supplementary material for: Duration of mRNA vaccine protection against SARS-CoV-2 Omicron BA.1 and BA.2 subvariants in Qatar
Source: Nat Commun. 2022 Jun 2;13:3082. doi: 10.1038/s41467-022-30895-3 (PMC9163167; doi:10.1038/s41467-022-30895-3)
Supplement: Supplementary file 2 — Reporting Summary [file 41467_2022_30895_MOESM2_ESM.pdf]

nature research

Corresponding author(s): Laith J Abu-Raddad

Last updated by author(s): May 9, 2022

Reporting Summary

Nature Research wishes to improve the reproducibility of the work that we publish. This form provides structure for consistency and transparency in reporting. For further information on Nature Research policies, see our Editorial Policies and the Editorial Policy Checklist.

Statistics

For all statistical analyses, confirm that the following items are present in the figure legend, table legend, main text, or Methods section.

n/a	Confirmed	
☐	☒	The exact sample size (*n*) for each experimental group/condition, given as a discrete number and unit of measurement
☐	☒	A statement on whether measurements were taken from distinct samples or whether the same sample was measured repeatedly
☐	☒	The statistical test(s) used AND whether they are one- or two-sided<br>*Only common tests should be described solely by name; describe more complex techniques in the Methods section.*
☐	☒	A description of all covariates tested
☐	☒	A description of any assumptions or corrections, such as tests of normality and adjustment for multiple comparisons
☐	☒	A full description of the statistical parameters including central tendency (e.g. means) or other basic estimates (e.g. regression coefficient) AND variation (e.g. standard deviation) or associated estimates of uncertainty (e.g. confidence intervals)
☒	☐	For null hypothesis testing, the test statistic (e.g. *F*, *t*, *r*) with confidence intervals, effect sizes, degrees of freedom and *P* value noted<br>*Give P values as exact values whenever suitable.*
☒	☐	For Bayesian analysis, information on the choice of priors and Markov chain Monte Carlo settings
☒	☐	For hierarchical and complex designs, identification of the appropriate level for tests and full reporting of outcomes
☐	☒	Estimates of effect sizes (e.g. Cohen's *d*, Pearson's *r*), indicating how they were calculated

Our web collection on statistics for biologists contains articles on many of the points above.

Software and code

Policy information about availability of computer code

Data collection	No software was used for data collection
Data analysis	Analyses were conducted in STATA/SE 17.0. The commands/code are accessible using URL: https://github.com/IDEGWCMQ/VE-Code/blob/main/VE%20Code.do

For manuscripts utilizing custom algorithms or software that are central to the research but not yet described in published literature, software must be made available to editors and reviewers. We strongly encourage code deposition in a community repository (e.g. GitHub). See the Nature Research guidelines for submitting code & software for further information.

Data

Policy information about availability of data

All manuscripts must include a data availability statement. This statement should provide the following information, where applicable:

- Accession codes, unique identifiers, or web links for publicly available datasets
- A list of figures that have associated raw data
- A description of any restrictions on data availability

The dataset of this study is a property of the Qatar Ministry of Public Health that was provided to the researchers through a restricted-access agreement that prevents sharing the dataset with a third party or publicly. The data are available under restricted access for preservation of confidentiality of patient data. Access can be obtained through a direct application for data access to Her Excellency the Minister of Public Health (https://www.moph.gov.qa/english/OurServices/eservices/Pages/Governmental-Health-Communication-Center.aspx). The raw data are protected and are not available due to data privacy laws. Data were available to authors through .csv files where information has been downloaded from the CERNER database system (no links/accession codes were available to authors). Aggregate data are available within the manuscript and its Supplementary information.

Field-specific reporting

Please select the one below that is the best fit for your research. If you are not sure, read the appropriate sections before making your selection.

☒ Life sciences ☐ Behavioural & social sciences ☐ Ecological, evolutionary & environmental sciences

For a reference copy of the document with all sections, see nature.com/documents/nr-reporting-summary-flat.pdf

Life sciences study design

All studies must disclose on these points even when the disclosure is negative.

Sample size

COVID-19 laboratory testing, vaccination, clinical infection data, and related demographic details were extracted from the integrated nationwide digital-health information platform that hosts the national, federated SARS-CoV-2 databases. These databases are complete and have captured all SARS-CoV-2-related data since epidemic onset. The data is based on a national cohort that includes every single individual tested using PCR in Qatar. Sample size varied depending on the definition used for cases [PCR-positive swabs for the Omicron variant that was collected because of symptoms compatible with a respiratory tract infection (any Omicron infection, or BA.1 Omicron infection, or BA.2 Omicron infection, depending on the analysis), as well as severe, critical, or fatal COVID-19 disease due to Omicron infection] and controls (PCR-negative swabs). Cases and controls were exact-matched by sex, 10-year age group, nationality, and calendar week of PCR test. The ratio of matching in each analysis was determined based on available number of cases and controls (Figure 2). In each analysis for a specific time-since-vaccination stratum, we included only those vaccinated in this specific time-since-vaccination stratum and those unvaccinated (our reference group). Thus, the number of cases (and controls) varied across time-since-vaccination analyses. Given that the sample sizes were based on national cohorts with only individuals that do not fit the eligibility criteria excluded, the sample size for each sub-study can be considered sufficient. Detailed sample sizes can be found in Figure 2 and Tables 1 and 2.

Data exclusions

Exclusion criteria were specified for cases and controls in each study group a priori. Cases and controls were excluded if they were not tested for clinical suspicion or if they had a vaccination record with ChAdOx1 nCoV-19. Additionally, cases and controls were excluded if they received a different vaccine from that under study. Only the first PCR-positive test with confirmed Omicron infection during the study, December 23, 2021 to February 28, 2022, was included for each case, whereas all PCR-negative tests during the study were included for each control.

Replication

For replication, three additional independent analyses were conducted to estimate each measure of vaccine effectiveness after 1) adjusting for prior infection and health worker status in conditional logistic regression analyses, 2) restricting the analysis to exclude children <12 years of age, and 3) restricting the analysis to individuals <20 years of age. All analyses confirmed/reproduced estimates of vaccine effectiveness obtained in the main analysis. In addition, a case-only analysis was conducted to examine differential waning for BA.1 versus BA.2 by comparing odds of BA.2 infection to odds of BA.1 infection among those vaccinated, with exposure being time since vaccination. All analyses confirmed/reproduced estimates of vaccine effectiveness obtained in the main analysis.

Randomization

Not applicable as this is an observational case-control study where individuals are aware of both their infection status and their vaccination status. However, to ensure control for confounding, cases and controls were exact-matched by sex, 10-year age group, nationality, and calendar week of PCR test. The ratio of matching in each analysis was determined based on available number of cases and controls (Figure 2). To ensure that vaccine effectiveness estimates were not biased, conditional logistic regression analyses were applied and sensitivity analyses were conducted by additionally adjusting for prior infection and health worker status in conditional logistic regression analyses.

Blinding

Not applicable as this is an observational case-control study where individuals are aware of both their infection status and their vaccination status.

Reporting for specific materials, systems and methods

We require information from authors about some types of materials, experimental systems and methods used in many studies. Here, indicate whether each material, system or method listed is relevant to your study. If you are not sure if a list item applies to your research, read the appropriate section before selecting a response.

Materials & experimental systems

n/a	Involved in the study	
☒	☐	Antibodies
☒	☐	Eukaryotic cell lines
☒	☐	Palaeontology and archaeology
☒	☐	Animals and other organisms
☐	☒	Human research participants
☒	☐	Clinical data
☒	☐	Dual use research of concern

Methods

n/a	Involved in the study	
☒	☐	ChIP-seq
☒	☐	Flow cytometry
☒	☐	MRI-based neuroimaging

Human research participants

Policy information about studies involving human research participants

Population characteristics	The demographic characteristics of the different study populations can be found in Tables 1 and 2.
Recruitment	This is a retrospective study where COVID-19 laboratory testing, vaccination, clinical infection data, and related demographic details were extracted from the integrated nationwide digital-health information platform that hosts the national, federated SARS-CoV-2 databases. These databases are complete with no missing information for PCR testing, COVID-19 vaccinations, COVID-19 hospitalizations, and basic demographic details, and have captured all SARS-CoV-2-related data since epidemic onset. Cases and controls were defined based on each analysis for these data. Cases were defined as a PCR-positive swab for the Omicron variant that was collected because of symptoms compatible with a respiratory tract infection (any Omicron infection, or BA.1 Omicron infection, or BA.2 Omicron infection, depending on the analysis), as well as severe, critical, or fatal COVID-19 disease due to Omicron infection, while controls were defined as a PCR-negative swab. Classification of COVID-19 case severity (acute-care hospitalizations), criticality (ICU hospitalizations), and fatality, followed the World Health Organization guidelines, and assessments were made by trained medical personnel using individual chart reviews. All records of PCR testing for those vaccinated and unvaccinated during the study duration were examined.
Ethics oversight	The study was approved by the Hamad Medical Corporation and Weill Cornell Medicine-Qatar Institutional Review Boards with waiver of informed consent.

Note that full information on the approval of the study protocol must also be provided in the manuscript.
